# *Plasmodium vivax* Infection in Multiple Family Members in Texas, USA

**DOI:** 10.1155/2019/8568710

**Published:** 2019-06-09

**Authors:** Roukaya Al Hammoud, James R. Murphy, Michael L. Chang

**Affiliations:** Department of Pediatrics, Division of Pediatric Infectious Diseases, The University of Texas Health Science Center at Houston, McGovern Medical School, Houston, TX 77030, USA

## Abstract

We report a cluster of 6 pediatric residents of Houston, Texas, USA, who presented with *Plasmodium vivax* infection within an eight-week period. All had immigrated to the United States from Afghanistan within the previous year. The clustering raises the possibilities of local mosquito vectored infection and/or synchronous relapses. Molecular typing and local mosquito testing are crucial in delineating the source of similar clusters in nonendemic regions. Single-dose hypnozoite eradication treatment may be considered in emigrating children to malaria nonendemic countries.

## 1. Introduction

*Plasmodium vivax* (*P. vivax*) is the most widespread human malaria with 2.5 billion people at risk for infection [1]. In the United States (US), residents visiting Asia are at highest risk of returning with this infection. While clustering is common in endemic regions, imported *P. vivax* clustering among immigrant family members in the US has not been described. We report a cluster of six pediatric patients who were recent immigrants from Afghanistan diagnosed with vivax malaria within a 2-month period centered around 8 months after immigration. We report this cluster to remind practitioners that some strains of vivax malaria may become symptomatic (from relapse or long incubation period) many months after an immigrant has arrived to the United States. This cluster also raises the question of a potential benefit from screening or presumptive treatment of *P. vivax* in asymptomatic immigrants from endemic regions arriving to areas with transmission competent *Anopholene* mosquito vectors, similar to the current guidelines in those departing from sub-Saharan Africa [2, 3].

## 2. Case Presentation

A 7-year-old previously healthy girl (patient 1, family A) presented to a children's hospital in Houston, Texas, in the spring of 2017 (day 1) with symptomatic vivax malaria. On day 5, patient 2, the twin brother of patient 1, presented with symptomatic vivax malaria. Subsequently, on day 9, 15, 41, and 55, patient 3, patient 4, patient 5, and patient 6, respectively, sought medical care for the same diagnosis. Diagnoses were made via microscopic examination of blood smears in our institution's laboratories.  Table 1 summarizes demographic, clinical, and laboratory characteristics of the patients. The patients belong to 2 related families (families A and B; the fathers are brothers) who live in 2 different apartment units on the same floor of the same residential complex. Both families emigrated from the temperate Jowzjan province in Northern Afghanistan, a country where vivax malaria is endemic (70–95%), even at high altitudes [4]. Patients were treated in Afghanistan for symptomatic vivax malaria with presumed chloroquine but the exact dates of their treatment could not be recalled by the parents. None of the patients received primaquine in the past. Patients arrived in Houston between 6 and 10.5 months prior to presentation with symptomatic vivax infection (Table 1). Both families reported exposure to mosquitoes in Houston. The 6 patients were initially treated by emergency physicians with atovaquone/proguanil as malaria speciation was not readily available. Patient 5 had an unusual high parasitemia (5%) and had no further confirmation of her results. All patients completely recovered after treatment and received primaquine as a hypnozoite-eradicating treatment.

## 3. Discussion

We report a cluster of vivax infections in 6 related children living in close proximity in a region considered free from endemic malaria transmission. While hypnozoite reactivation is the most probable explanation in these emigrating patients who had not previously received hypnozoite-eradicating treatment, the clustering did raise concerns for possible local mosquito-borne transmission of *P. vivax* mainly with the patients' reported history of recent mosquito contact. In the distant past, *P. vivax* was endemic in the Houston region. Mosquito transmission of *P. vivax* was reported in 1994, and *P. vivax*-competent *Anopheles* are endemic to Houston [5]. All 6 of our patients were reported to local health authorities. An epidemiological investigation was not initiated, and molecular typing and local mosquito testing were not feasible at our institution's clinical laboratories. There were no known secondary cases that occurred outside these 2 families per local health department authorities. Also, the cluster was recognized at its later stage, which made further testing impossible, given the unavailability of the patients' samples. The onset of illness in patients 1, 2, 3, and 4 was too close for *P. vivax* to complete its cycle in the mosquito and its incubation period in patients. Based on the limited available data, there was no evidence of local mosquito transmission. Less common sources of malaria as airport malaria and blood transfusion were excluded and unlikely to explain a cluster.

In nonendemic regions, familiarity with the different phenotypes may be limited, and vivax malaria presenting outside of endemic areas is often considered a single homogeneous species with providers failing to recognize the substantial phenotypic variation between *P. vivax* strains. [6] Tropical strains of *P. vivax* usually relapse at 3 weeks intervals if treated early with antimalarials. In contrast, temperate region “long-latency” *P. vivax*, considered prevalent in Afghanistan, has long incubation period (8–10 months) or extended period between malaria illness and first relapse (8–10 months) [6, 7]. As such, this cluster may be primary presentation for “hibernans” vivax malaria or long-latency relapse. It is likely that “preprogrammed” hypnozoites with biologic clock decide when they resume development, causing activation and relapse. It is both plausible and probable that our family members were primarily infected with the same “strain” of vivax malaria in Afghanistan, which could explain close incubation periods. Another factor that can influence incubation period is initial inoculum of sporozoites. The higher the dose of sporozoites inoculated in patients by mosquito, the shorter the latency period between malaria illness and first relapse and the more frequent relapses. [6] Immunity may also influence incubation period and latency. In our patients, unfortunately it was difficult to clarify through history taking, when the previous malaria illnesses occurred in Afghanistan. However, since they reported prior antimalarial therapy (though without primaquine), we suspect this to be a first relapse for a long-latency strain, as there were no reported malaria illnesses after their immigration to the US prior to this presentation.

Hypnozoites can remain dormant for months or even years. The exact mechanism of hypnozoite dormancy and activation is not well understood with multiple factors involved. For “long-latency” temperate vivax strains, subsequent relapses after the first are shorter, usually weeks, and for unclear reasons, subsequent relapses tend to occur periodically [6]. There are many concepts related to activation of hypnozoites and relapse. Activation of hypnozoites could also be induced by a new malarial febrile illness caused by a new plasmodium inoculation. It was also suggested that uninfected mosquito biting can induce hypnozoite activation, possibly via sensing of a mosquito protein [6, 8]. Hypnozoites can adapt to the phenology of local vectors, and activation would coincide mosquito season. For example, imported *P. vivax* reactivation cases in the United Kingdom were reported to be limited to the summer months irrespective of the time of initial infection. [9] As noted in these previous studies, our patients also presented at the onset of the rainy season and usual timing of mosquito population increases. Household clustering is common in malaria-endemic areas, likely secondary to simultaneous transmission, but few clusters of imported *P. vivax* infection have been reported in nonendemic areas [10, 11]. Imported *P. vivax* clustering among immigrant family members in the US has not been described. We present this case to remind providers in nonendemic regions that immigrants from some vivax malaria-endemic regions may have long incubation periods and long-latency periods prior to development of symptoms. Given the large population of *Anopholene* mosquitoes in our area, providers need to remember that treatment should include primaquine to eradicate hypnozoites (as in our patients) and also focus on minimizing transmission via mosquito repellent and good mosquito control in the local environment.

Finally, all malaria cases and especially those representing the unique circumstance of a cluster should be promptly reported to health departments so that the epidemiology of the disease can be identified. Optimally, molecular typing of clinical specimens and local mosquito testing for *Plasmodium* species will give more evidence delineating the origin of similar clusters. As migration, climate, and environmental factors change, immigrants, especially children, from regions of high prevalence of *P. vivax* could be screened (as immigrants from sub-Saharan Africa are for falciparum) and might be considered for single-dose 8-aminoquinoline treatment to prevent local transmission in nonendemic areas with *Anopholene* vectors [12].

## Figures and Tables

**Table 1:** Clinical and laboratory characteristics of the patients.

	Patient 1	Patient 2	Patient 3	Patient 4^*∗*^	Patient 5	Patient 6
Age (years)	7	7	11	2.8	5	6
Gender	Female	Male	Male	Male	Female	Female
Family	A	A	B	A	B	B
Relation to patient 1		Twin brother	Cousin	Sibling of patient 1 and 2	Sibling of patients 3	Sibling of patients 3 and 5
Day of presentation	Day 1	Day 5	Day 9	Day 15	Day 41	Day 55
Presenting symptoms	Fever, chills, headache, and myalgia	Fever, myalgia, and fatigue	Fever, chills, nausea, and fatigue	Fever, chills, emesis, headache, and myalgia	Fever, fatigue, emesis, and abdominal pain	Fever, nausea, emesis, headache, and cough
Organomegalies	None	None	None	None	None	Mild splenomegaly
Hemoglobin (g/dl)	9.4	11.2	13.7	11.3	11.3	11.2
Platelets (per mm^3)^	51,000	87,000	152,000	99,000	108,000	69,000
Initial parasitemia (%)	0.25	0.11	1.3	2.4	5	1.2
Period between immigration to US and illness	∼6 months	∼6 months	∼9 months	∼6 months	∼10 months	∼10.5 months
Previous malaria history	Yes	Yes	Yes	Yes	Yes	Yes
Previous blood transfusion or travel outside Houston since immigration	No	No	No	No	No	No
Parasitemia (%); time after treatment	0.1, 3 days	Not done	Negative, 7 days	0.5, 2 days; negative, 65 days	Negative, 5 days	0.01, 25 days

^*∗*^All patients are previously healthy except patient 4 (history of idiopathic thrombocytopenic purpura).
